# Comparison of the effects of indoor and outdoor exercise on creativity: an analysis of EEG alpha power

**DOI:** 10.3389/fpsyg.2023.1161533

**Published:** 2023-07-18

**Authors:** Tsukasa Kimura, Teruhiro Mizumoto, Yuusuke Torii, Masumi Ohno, Teruo Higashino, Yasushi Yagi

**Affiliations:** ^1^SANKEN (The Institute of Scientific and Industrial Research), Osaka University, Osaka, Japan; ^2^Graduate School of Information Science and Technology, Osaka University, Osaka, Japan; ^3^ASICS Corporation, Hyogo, Japan; ^4^Faculty of Engineering, Kyoto Tachibana University, Kyoto, Japan

**Keywords:** creativity, exercise, electroencephalogram (EEG), alternative uses test (AUT), flow state scale (FSS)

## Abstract

Previous research finds that natural environments and exercise enhance creativity. In this within-subjects design study, we examined the influence of outdoor exercise that combined a natural environment with exercise on creativity compared to an indoor exercise control condition by analyzing cognitive activities related to creativity. The participants performed an Alternative Uses Test (AUT), in which ordinary objects are presented to the participants (e.g., a brick), to prompt as many ideas for alternative uses as possible, which are transformed into a creativity score, after indoor running and outdoor running. During the test, brain activity was recorded using electroencephalography (EEG) and a short version flow state scale (FSS) was completed after the experiment. Results showed that while AUT scores did not significantly differ between conditions, alpha band activity at the parietal occipital region involved in divergent creativity increased during the AUT after outdoor exercise while it did not during the AUT after indoor exercise. In addition, FSS scores for positive emotional experience and absorption were higher after outdoor exercise than after indoor exercise. Our results from the FSS suggest that exercise in a natural environment is perceived subjectively differently from indoor exercise, participants report greater experiences of flow compared to indoor exercise, and the EEG measures objectively indicate enhanced cognitive activity in a creativity task after outdoor exercise. This study suggests that outdoor exercise increases neuronal activity in brain regions related to creativity. Further research is needed to understand how this can lead to increased creativity.

## 1. Introduction

Creativity is an important, complex, and multifaceted concept for work, art, society, and many other fields (e.g., Hennessey and Amabile, 2010; Zhou and Hoever, 2014; Dietrich, 2019). Currently, creativity has been examined using various methodologies, e.g., psychology, neuroscience, and many other fields, and these studies reported that creativity influences various levels of human life, such as individual cognition, work engagement, and culture (e.g., Fink et al., 2009; Hennessey and Amabile, 2010; Dietrich, 2019; Khalil et al., 2019). In addition, the development of artificial intelligence technology can automate simple tasks, and humans are expected to engage in creative tasks (Corazza, 2017). Therefore, there is increasing interest in how to enhance creativity and promote activities in each field.

As the way to enhance creativity, previous studies have reported findings involving the work environment and the effect of exercise. Regarding the work environment, some studies reported that distractions such as background noise, a disorderly environment and the natural environment increased creativity (e.g., Atchley et al., 2012; Mehta et al., 2012; Vohs et al., 2013; Chulvi et al., 2020). In particular, the enhancement of creativity by spending time in a natural environment has been explained with the Attention Restoration Theory (ART) framework of the effect of the natural environment on a person’s psychological state (e.g., Kaplan and Kaplan, 1989; Kaplan, 1995; Kaplan and Berman, 2010). In ART, it is interpreted that the natural environment separates one’s attention from one’s work (i.e., distracting from the work) and stops the consumption of attention by the work, thereby benefitting various aspects of cognitive processing. To enhance creativity, it is thought to be important to be in a diffuse attention state in which one is not focused on specific work and tasks but also pays attention to “task-irrelevant” information (e.g., Williams et al., 2018; Zmigrod et al., 2019). Taking these studies together, it is interpreted that the natural environment distracts the attention from the task and that the attention is focused more on task-irrelevant information; thereby, the natural environment enhances creativity. With respect to physical exercise, previous studies reported that moderate intensity exercise, such as running and cycling without maximum effort, increased creativity (e.g., Netz et al., 2007; Oppezzo and Schwartz, 2014; Aga et al., 2021). These enhancement effects on creativity are interpreted to occur because exercise influences subjective cognitive status through neurotransmitters such as serotonin, dopamine, and endocannabinoids (Aga et al., 2021).

As a consequence, it might be possible that combining these natural environments with exercise (i.e., outdoor exercise) enhances creativity. Previous studies reported that outdoor exercise enhances the increase in positive emotion, decrease in negative emotion, and promotion of attention compared to indoor exercise (e.g., Focht, 2009; Thompson Coon et al., 2011; Rogerson et al., 2016). Based on the ART, considering the effect of outdoor exercise on creativity, it is possible that the natural environment enhances diffuse attention during exercise and improves creativity (e.g., Williams et al., 2018; Zmigrod et al., 2019). In addition, the Stress Reduction Theory (SRT) framework showed that light physical activity is associated with decomposition of cortisol in an outdoor educational setting while this is not the case in an indoor setting (Becker et al., 2019), and that this effect might have an impact on pupils’ cerebral maturation (Dettweiler et al., 2023). A previous study has reported the influence of outdoor exercise for creativity (Oppezzo and Schwartz, 2014); however, it offered insufficient comparison with indoor exercise. Moreover, if outdoor exercise enhances creativity, it might influence the cognitive activities involved in creativity. Traditionally, creativity has been measured by the two types of thinking: divergent and convergent. Divergent thinking is related to the ability to generate multiple solutions by novel perspectives and ideas. For example, in the Alternative Uses Test (AUT), a common test for measuring divergent thinking, participants are presented with common objects (e.g., a brick) and asked to describe as many uncommon and unique uses for those objects as possible (e.g., use as a training barbell) within a time limit (Guilford, 1950; Guilford, 1967). Convergent thinking is related to combining different ideas to determine a single and correct solution to a problem. For example, in the Remote Associates Test (RAT), a common test for measuring convergent thinking, participants are presented with three words (e.g., “cottage,” “Swiss,” and “cake”) and asked to provide an answer associated with these words (“cheese”) within a time limit (Mednick, 1962; Bowden and Jung-Beeman, 2003). Previous studies have reported an increase in alpha band power activity (8–13 Hz) of an electroencephalogram (EEG) at the frontal and parietal-occipital region in relation to creativity, especially divergent thinking (e.g., Martindale and Hasenfus, 1978; Mölle et al., 1999; Fink et al., 2009; Fink and Benedek, 2014). This increase in alpha band power activity is interpreted to reflect not simply the processing of task-relevant information but the retrieval of stored knowledge and the recombination of stored memory elements to enhance creative activity (Fink and Benedek, 2014). If outdoor exercise enhances creativity, it might be possible that the alpha band power activity during the creativity task increases after outdoor exercise compared to after indoor exercise. This is a plausible hypothesis, considering that a previous study reported the enhancement of alpha band power activity in natural outdoor environments (Grassini et al., 2022).

To test these hypotheses regarding the effect of outdoor exercise on creativity, this study compared the effects of indoor and outdoor exercise on creativity and its cognitive activities. The participants participated in the experiment for 10 days. On days 1 and 2, the baseline for creativity (i.e., divergent thinking) before the exercise on days 3 to 5 was measured by AUT and by EEG during the AUT, and a short version of the flow state scale (FSS; Jackson and Marsh, 1996) was used to measure the subjective sense of control (SC), positive emotional experience (PEE), and absorption by concentrating (AC) on the AUT. This scale had been used in a previous study to measure the effect of not only exercise but also physical and cognitive tasks (Yoshida et al., 2013). The previous studies reported that flow state relates to exercise (Wollseiffen et al., 2016) and creativity (Jaque et al., 2020). After the day 2 experiment, the moderate intensity running exercise for each participant was measured by the rating of perceived exertion (RPE) using a treadmill in an indoor room (Borg, 1970). On days 3 to 5, participants performed either indoor (i.e., running on a treadmill) or outdoor (i.e., running in a natural park) exercise of moderate intensity for 30 min, and after the running, their AUT score, EEG during the AUT, and FSS score were measured. On days 6 and 7, a baseline of before the exercise on day 8 to 10 for AUT, EEG during the AUT, and FSS were measured again without the exercise. On days 8 to 10, participants performed the other exercise they did not perform on days 3 to 5, and after running, their AUT score, EEG during the AUT, and FSS score were measured. Therefore, participants who performed an indoor exercise (outdoor exercise) on days 3–5 performed an outdoor exercise (indoor exercise) on days 8–10. The differences between the baseline and AUT, EEG, and FSS after each exercise were calculated to compare the effects of indoor and outdoor exercise on creativity. If outdoor exercise enhances the neuronal activities involved in creativity, we hypothesized that the alpha band power activity of the EEG after the outdoor exercise would increase compared with that after the indoor exercise (e.g., Martindale and Hasenfus, 1978; Mölle et al., 1999; Fink et al., 2009; Fink and Benedek, 2014). Moreover, although creativity scores are believed to be higher in the outdoor condition, the decreasing effect of repeated testing may offset the anticipated increase in creativity scores in an experimental design that counterbalances the order of outdoor/indoor testing (serial order effect in divergent thinking; Beaty and Silvia, 2012). Therefore, we hypothesized that the AUT scores of the first day of outdoor/indoor exercise would remain at or decrease from the baseline, that these scores would not be increased through the experience of exercising outdoors/indoors three times, and that no significant difference between AUT scores for the outdoor and indoor conditions would be observed. Furthermore, we hypothesized that the FSS scores after the outdoor exercise would increase compared with those after the indoor exercise if outdoor exercise enhances creativity (Wollseiffen et al., 2016; Jaque et al., 2020).

## 2. Materials and methods

### 2.1. Participants

Twenty undergraduate and graduate students (10 females, 10 males; 19–29 years of age) participated in the within-subjects design experiment. Power analysis was conducted using R and the pwr package (Champely et al., 2018). With this sample size, the effect size for the two-way repeated measures analysis of variance (ANOVA) on Δ alpha power (ln(μV^2^)) among two conditions (indoor and outdoor) and three periods (first, second, and third day) that were the main factors of this study was f2 = 0.26 at a significance level of *α* = 0.05 and power of 1 – *β* = 0.80, which was a medium effect (Cohen, 1992). All participants were right-handed, according to their self-report, and had normal or corrected-to-normal vision. This experiment was approved by the Graduate School of Information Science and Technology’s Research Ethics Review Board under Osaka University Regulations. Written informed consent was obtained from all participants, and their rights as experimental subjects were protected. All methods were performed in accordance with the relevant guidelines and regulations.

### 2.2. Stimulus and equipment

#### 2.2.1. Apparatus

In the experimental room, the instruction and AUT were presented on a 13.3-inch LCD monitor of a laptop computer ROG Flow X13 (ASUS Inc., Taiwan). The visual angle of each character was 0.5° by 0.5° from an observing distance of 60 cm. The presentation of instruction and AUT were controlled with VBA for applications of Microsoft office Excel (Microsoft Inc., U.S.A.). The FSS was presented on a 10.2-inch LCD monitor of an iPad (Apple Inc., U.S.A.). The visual angle of each character was 0.5° by 0.5° from an observing distance of 60 cm. The measurement of ratings of perceived exertion and performance of indoor exercise were done using the treadmill SKILLRUN and MYRUN (Technogym, Italy). The amount of indoor exercise and outdoor exercise were measured by Fitbit Versa 3 (Fitbit Inc., U.S.A.).

#### 2.2.2. Alternative Uses Test (AUT)

Creativity was measured by the AUT because previous research reported that outdoor exercise increased divergent creativity but not convergent creativity (Oppezzo and Schwartz, 2014). The AUT had been used in a previous study as a divergent creativity task. In the AUT, participants were presented with common objects (e.g., brick) and asked to describe as many uncommon and unique uses of those objects as possible (e.g., use as a training barbell) within a time limit. In our study, participants performed the AUT using a keyboard for 5 min per object and performed it for three objects per day (i.e., 15 min). Participants participated for 10 days; therefore, in this experiment participants gave answers for thirty different objects. The order of presentation of objects was randomized. Each day’s creativity scores were calculated by averaging the score of three objects. Fluency, flexibility, and originality were calculated to generate the creativity score. Fluency was the number of answers describing uncommon uses, flexibility was the number of categories of answers, and originality was the rareness of the answers. Three researchers familiar with psychological experiments calculated these creativity scores independent of these authors. They were not the authors’ colleagues or collaborators, and they had no conflicts of interest. Fluency was scored after three researchers independently deleted similar answers for each object (Guilford, 1967). Flexibility was scored as the mean number of categories of answers after three researchers independently summarized similar categories of answers (Guilford, 1967). Originality was scored as the mean score of 1 to 5 independently scored by three researchers for each fluency answer (Silvia et al., 2008). The degree of agreement for the AUT scores of the three researchers was evaluated by intraclass correlation coefficient (ICC(2,1)) using R and the psych package (Revelle, 2017).

#### 2.2.3. Flow state scale (FSS)

A short version of the flow state scale (FSS) was used to measure the subjective sense of control (SC), positive emotional experience (PEE), and absorption by concentrating (AC) on the AUT. This scale had been used in a previous study to measure the effect of not only exercise but also physical and cognitive tasks (Yoshida et al., 2013). The participants answered 14 items on a 7-point scale (scores range from 1 to 7) *via* iPad, score 1 meant strongly disagree and score 7 meant strongly agree. Answer form of FSS automatically popped up when a participant launches an iPad, displaying 14 items within one page of the form, and the participant responded by touching the appropriate part of the Likert scale. These items consisted of three factors of SC, PEE, and AC. A low score means low sense of control, positive emotional experience, and absorption by concentrating. The internal consistency for each subscale of the FSS was evaluated by Cronbach’s alpha using R and the psych package (Revelle, 2017).

#### 2.2.4. Indoor exercise and outdoor exercise

**Figure 1:**
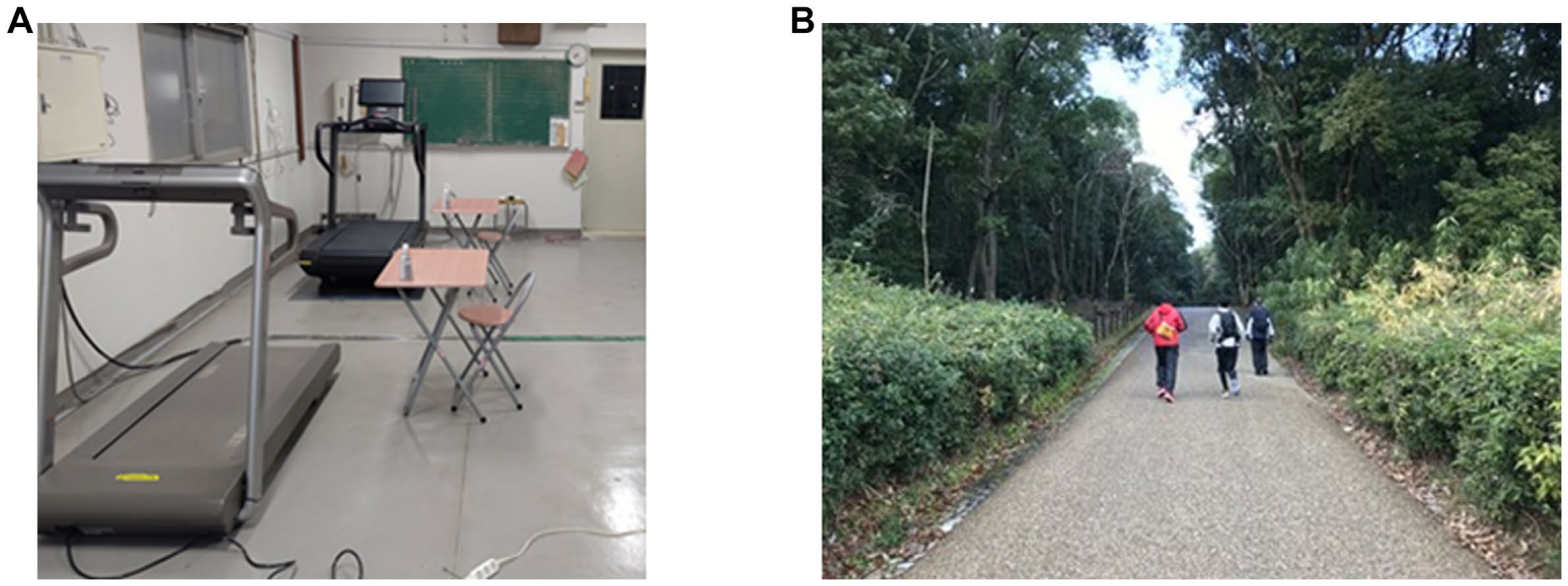
The environment of **(A)** indoor exercise (running on a treadmill in a room) and **(B)** outdoor exercise (running in a forest park).

To decide the amount and intensity of exertion for the performance of indoor and outdoor exercise, the RPE and heart rate (HR) were measured by subjective rating and Fitbit Versa 3 when participants ran on a treadmill indoors after the day 2 experiment (Borg, 1970, 1982). In the measurement of RPE, participants were instructed regarding the exertion level of RPE, that is, the rating 6, 7.5, 9, 11, and 14 meant as no exertion at all, extremely light, very light, light, and somewhat hard exertion. After the instruction, participants were asked to run for 1 min on an indoor treadmill set at 5 km/h and to rate their RPE. If this rating was less than 14, the treadmill speed was increased by 1 km/h and participants answered the RPE again after the running for 1 min. This procedure was repeated until the RPE rating reached 14, and the running speed and HR were recorded. Each participant was told their running speed and the HR of their RPE rating at 11 to 14, and were instructed to run for 30 min at this range of running speed and HR during indoor and outdoor exercise on days 3 to 5 and 8 to 10 with monitoring by Fitbit Versa 3. The indoor exercise took place on a treadmill in a room and the outdoor exercise took place in a forest park (Figure 1).

#### 2.2.5. Procedure

**Figure 2:**
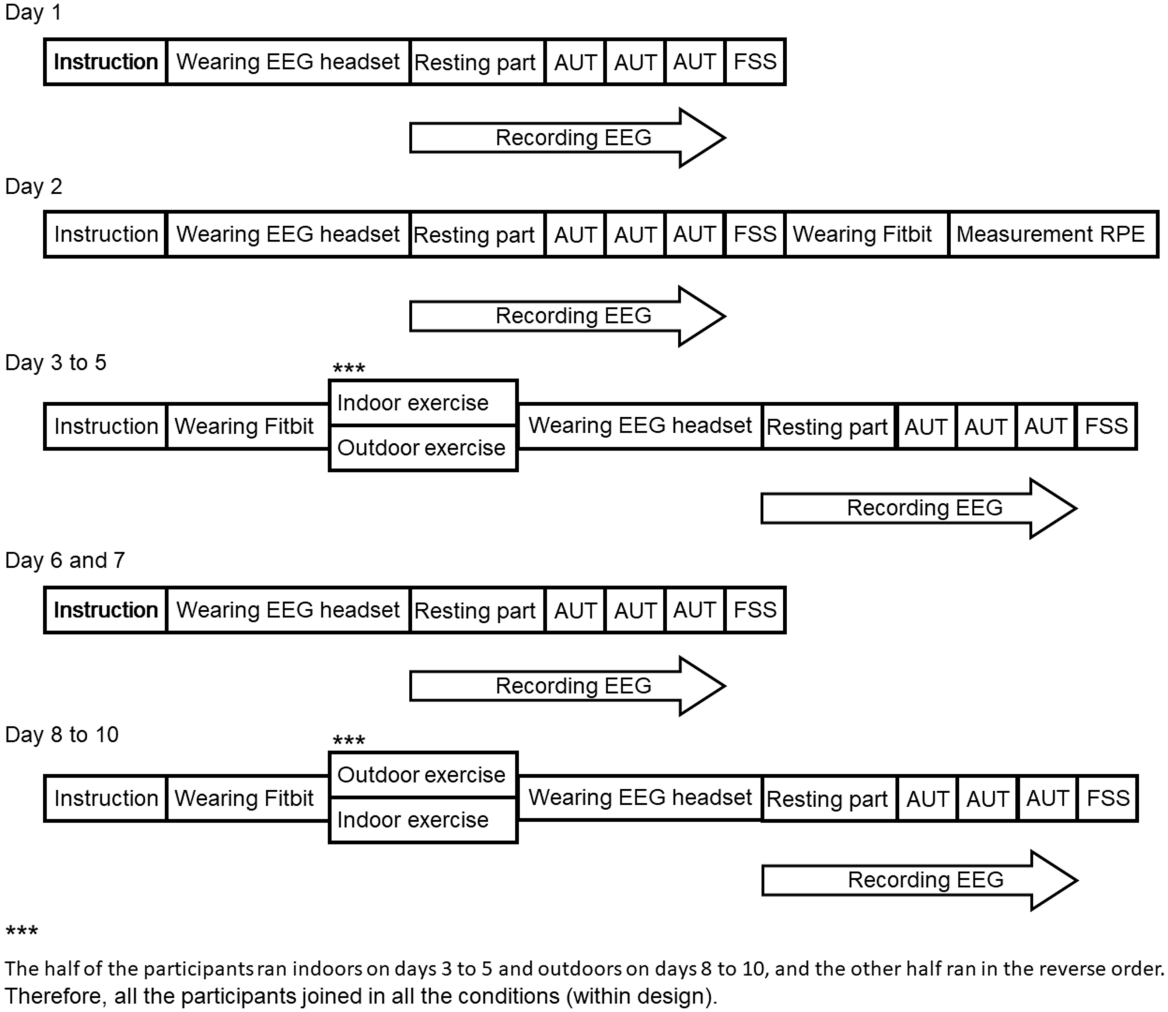
The procedures of the experiment each day.

This experiment was designed the within-subjects design, Figure 2 illustrates the experimental procedure. On days 1 and 2, the participants first performed the AUT. In the experimental room, participants sat in a chair with electrodes placed on their heads to record the EEG. They were asked not to move more than necessary to avoid artifacts in the physiological data. During the resting part, participants were asked to gaze at a black fixation cross in the center of a white background for 60 s. After the resting part, participants were presented with common objects, asked to describe as many uncommon and unique uses for those objects as possible within the time limit by using keyboard for 5 min per object, and performed this for the three objects. After the AUT, participants responded to the FSS, and the RPE and HR were measured by subjective rating and Fitbit Versa 3 when participants ran on the treadmill indoors after the day 2 experiment. On days 3 to 5, participants were told to run for 30 min indoors (treadmill in a room) or outdoors (forest park) at the running speed and HR of the RPE rating of 11 to 14. After running, participants performed the AUT and their EEG was recorded, and they responded to the FSS like they did on day 1. On days 6 and 7, participants performed the experiment like they did on day 1. On days 8 to 10, the participants performed the running indoors or outdoors that they did not perform on days 3 to 5, i.e., all participants experienced both types of running. The order of running was counterbalanced between participants such that half of the participants ran indoors on days 3 to 5 and outdoors on days 8 to 10, and the other half ran in the reverse order.

#### 2.2.6. Recording electroencephalogram (EEG)

EEG data were recorded by DSI-24 (Wearable Sensing, U.S.A.) using active dry electrodes at 19 sites (Fp1, Fp2, Fz, F3, F4, F7, F8, Cz, C3, C4, T3, T4, T5, T6, P3, Pz, P4, O1, and O2) according to the modified 10–20 System. In addition, electrodes were placed on both earlobes (A1 and A2) as the reference electrode, on AFz as the ground electrode, and on the Pz as the common mode follower electrode. The data from all channels were recorded using the DSI streamer (Wearable Sensing, U.S.A.). The electrode impedances were kept below 500 kΩ. A 0.003–150 Hz band-pass filter was used at recording. The sampling rate was 300 Hz.

#### 2.2.7. Data analysis

To analyze the scores of fluency and originality for AUT, each day’s score was averaged and the average score of the no exercise day was subtracted from the scores of exercise days (baseline correction; Δ fluency score, Δ flexibility score, and Δ originality score). For example, in the schedule of one participant, she/he participated in no exercise days on days 1 and 2, indoor exercise days on days 3–5, no exercise days on days 6 and 7, and outdoor exercise days on days 8–10. In this schedule, the average scores for days 1 and 2 without exercise were subtracted from the scores for the indoor exercise days (days 3–5), and the average scores for days 6 and 7 without exercise were subtracted from the scores for the outdoor exercise days (days 8–10). As with the scores of the AUT, the average scores of the no exercise days of the SC, PEE, and AC for FSS were subtracted from the scores of the exercise days (baseline correction; Δ SC score, Δ PEE score, and Δ AC score).

To analyze the EEG data, the EEGLAB toolbox (Delorme and Makeig, 2004) and ERPLAB toolbox (Lopez-Calderon and Luck, 2014) on MATLAB (MathWorks Inc) were used. Artifacts derived from eye movements and eye blinks were rejected using an automatic EEG artifact detector based on the joint use of spatial and temporal features (ADJUST) of the EEGLAB toolbox (Mognon et al., 2011). After artifact rejection, the EEG data were digitally band-pass filtered at 8–13 Hz (6 dB/octave; order: 5,000) using an IIR Butterworth analog simulation filter. Moreover, this voltage was squared and natural logarithms at the frontal area electrodes (F3, Fz, and F4) and at the parietal-occipital area electrodes (P3, P4, O1, Oz, and O2) were calculated to analyze the alpha band power as an index of divergent creativity. These electrodes were chosen according to previous studies (e.g., Fink et al., 2009; Fink and Benedek, 2014; Agnoli et al., 2020). These areas’ alpha band powers were averaged for the period of before the AUT (rest period; 1 min) and the period of the AUT (AUT section; 15 min), and the rest period was subtracted from the AUT period (baseline corrected alpha band power). As with the AUT and FSS scores, for the baseline corrected alpha band powers at the frontal area electrodes and parietal-occipital electrodes the average score of the no exercise days was subtracted from the scores of exercise days (Δ alpha band power).

In summary, the Δ AUT scores, Δ FSS scores, and Δ alpha band powers were summarized of two conditions (indoor exercise and outdoor exercise) and three times of experience of exercise (first, second, and third). For the experience of exercise, in the schedule of one participant, if she/he participated indoor exercise days on days 3–5, experience of indoor exercise a first meant on day 3, a second meant on day 4, and a third meant on day 5. In addition, she/he participated outdoor exercise days on days 8–10, experience of outdoor exercise a first meant on day 8, a second meant on day 9, and a third meant on day 10.

The Δ AUT scores, Δ FSS scores, and Δ alpha band powers were assessed with a two-way repeated measures ANOVA (two conditions (indoor exercise and outdoor exercise) and three times of experience of exercise (first, second, and third)). These ANOVAs were conducted by applying Greenhouse–Geisser corrections to the degrees of freedom (Greenhouse and Geisser, 1959) when Mauchly’s sphericity test was significant. The effect sizes have been indicated in terms of partial eta squared (η^2^_*p*_). *Post hoc* comparisons were made using Shaffer’s modified sequentially rejective multiple test procedure, which extends Bonferroni t tests in a stepwise fashion (Shaffer, 1986). The significance level was set at *p* < 0.05 for all statistical analyses. Moreover, these results were additionally analyzed by Bayesian ANOVAs and *post-hoc* *t*-tests (Rouder et al., 2017; Wagenmakers et al., 2018) using JASP version 0.17.1 (JASP Team, 2023). Three models—condition, experience of exercise, and interaction between condition and experience of exercise—were compared to the null model. We examined whether the alternative hypothesis (H1) is supported by examining Bayes factors (BF_10_). Bayes factors were interpreted in accordance with previous studies (Schönbrodt and Wagenmakers, 2018). Prior odds and alpha levels were corrected for multiple comparisons (Westfall et al., 1997).

## 3. Results

### 3.1. The amount of indoor and outdoor exercise

The amount of indoor and outdoor exercise was recorded by HR *via* Fitbit Versa 3 during each exercise. The mean HR during indoor exercise over 3 days was 158.53 b.p.m. (SE = 1.53) and during outdoor exercise over 3 days was 153.61 b.p.m. (SE = 1.49).

### 3.2. Δ AUT score

**Figure 3:**
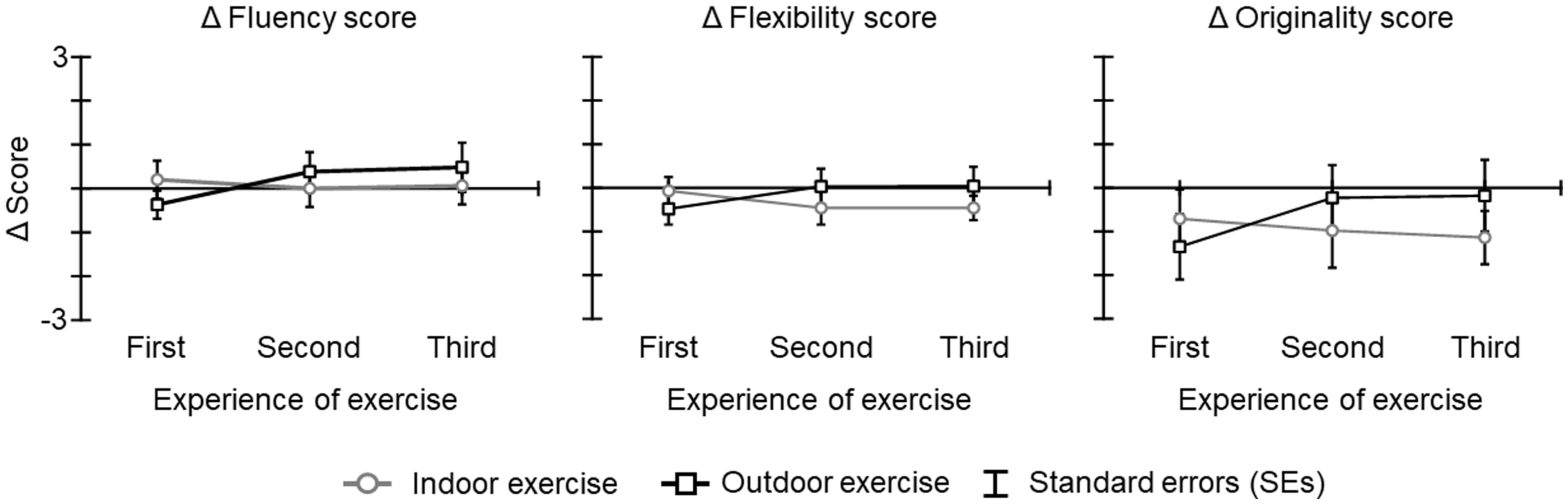
The Δ AUT scores for each condition and period. The error bars indicate the standard errors (SEs) of the means across participants.

The ICC(2,1) for the rating of each AUT score by three researchers exceeded 0.8 (fluency: ICC = 0.82; flexibility: ICC = 0.82; originality: ICC = 0.86). Figure 3 shows the Δ fluency score, Δ flexibility score, and Δ originality scores. A previous study reported that AUT scores decrease with repetition (serial order effect in divergent thinking; Beaty and Silvia, 2012). In fact, these scores were lower or comparable to the baseline and showed a floor effect from repeated testing during baseline. The results of the ANOVA and Bayesian ANOVA for all Δ scores revealed that the main effect of condition [Δ fluency score: *F*(1, 19) = 0.05, *p* = 0.829, η2 *p* = 0.003; BF_10_ = 0.308 indicating inconclusive evidence for alternative hypothesis; Δ flexibility score: *F*(1, 19) = 0.44, *p* = 0.515, η2 *p* = 0.021; BF_10_ = 0.359 indicating inconclusive evidence for alternative hypothesis; Δ originality scores: *F*(1, 19) = 0.60, *p* = 0.448, η2 *p* = 0.031; BF_10_ = 0.340 indicating inconclusive evidence for alternative hypothesis], the main effect of experience of exercise [Δ fluency score: *F*(2, 38) = 0.71, *p* = 0.468, ε = 0.785, η2 *p* = 0.036; BF_10_ = 0.182 indicating inconclusive evidence for alternative hypothesis; Δ flexibility score: *F*(2, 38) = 0.40, *p* = 0.933, ε = 0.802, η2 *p* = 0.002; BF_10_ = 0.123 indicating inconclusive evidence for alternative hypothesis; Δ originality scores: *F*(2, 38) = 0.31, *p* = 0.686, ε = 0.789, η2 *p* = 0.016; BF_10_ = 0.163 indicating inconclusive evidence for alternative hypothesis], and the interaction of condition and experience of exercise [Δ fluency score: *F*(2, 38) = 1.98, *p* = 0.160, ε = 0.842, η2 *p* = 0.095; BF_10_ = 0.043 indicating inconclusive evidence for alternative hypothesis; Δ flexibility score: *F*(2, 38) = 3.01, *p* = 0.066, ε = 0.919, η2 *p* = 0.131; BF_10_ = 0.056 indicating inconclusive evidence for alternative hypothesis; Δ originality scores: *F*(2, 38) = 1.93, *p* = 0.164, ε = 0.911, η2 *p* = 0.092; BF_10_ = 0.037 indicating inconclusive evidence for alternative hypothesis] were not significant and that Bayes factors were less than 1.

### 3.3. Δ FSS score

**Figure 4:**
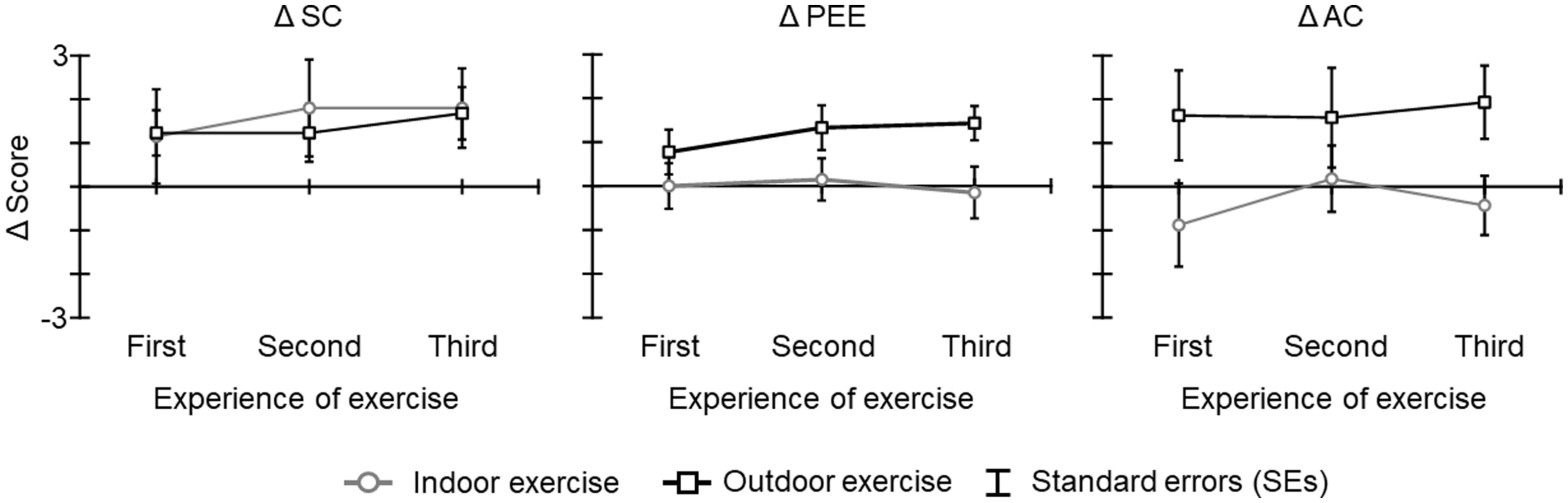
The Δ FSS scores for each condition and period. The error bars indicate the standard errors (SEs) of the means across participants.

The Cronbach’s alpha for each FSS score exceeded 0.7 (SC: *α* = 0.78; PEE: *α* = 0.88; AC: *α* = 0.87). Figure 4 shows the Δ SC score, Δ PEE score, and Δ AC scores. The results of the ANOVA and Bayesian ANOVA for Δ SC score revealed that the main effect of condition [*F*(1, 19) = 0.04, *p* = 0.834, η2 *p* = 0.002; BF_10_ = 0.405 indicating inconclusive evidence for alternative hypothesis], the main effect of experience of exercise [*F*(2, 38) = 0.50, *p* = 0.552, ε = 0.742, η2 *p* = 0.026; BF_10_ = 0.178 indicating inconclusive evidence for alternative hypothesis], and the interaction of condition and experience of exercise [*F*(2, 38) = 0.30, *p* = 0.735, *ε* = 0.973, η2 *p* = 0.015; BF_10_ = 0.015 indicating inconclusive evidence for alternative hypothesis] were not significant and that Bayes factors were less than 1. The results of the ANOVA and Bayesian ANOVA for Δ PEE scores revealed that the main effect of condition was significant [*F*(1, 19) = 5.41, *p* = 0.031, η2 *p* = 0.221; BF_10_ = 2.486 indicating anecdotal evidence for alternative hypothesis], and that the Δ PEE score of the outdoor exercise condition was higher than that of the indoor exercise condition (BF_10_, U = 14.191; posterior odds = 14.191). The main effect of experience of exercise [*F*(2, 38) = 0.65, *p* = 0.520, ε = 0.956, η2 *p* = 0.033; BF_10_ = 0.164 indicating inconclusive evidence for alternative hypothesis] and the interaction of condition and experience of exercise [*F*(2, 38) = 0.70, *p* = 0.490, ε = 0.937, η2 *p* = 0.036; BF_10_ = 0.103 indicating inconclusive evidence for alternative hypothesis] were not significant and that Bayes factors were less than 1. The results of the ANOVA and Bayesian ANOVA for Δ AC score revealed that the main effect of condition was significant [*F*(1, 19) = 5.95, *p* = 0.024, η2 *p* = 0.238; BF_10_ = 2.848 indicating anecdotal evidence for alternative hypothesis], and that the Δ AC score of the outdoor exercise condition was higher than that of the indoor exercise condition (BF_10_, U = 16.278; posterior odds = 16.278). The main effects of experience of exercise [*F*(2, 38) = 0.51, *p* = 0.545, ε = 0.730, η2 *p* = 0.026; BF_10_ = 0.136 indicating inconclusive evidence for alternative hypothesis] and the interaction of condition and experience of exercise [*F*(2, 38) = 0.46, *p* = 0.624, *ε* = 0.957, η2 *p* = 0.023; BF_10_ = 0.084 indicating inconclusive evidence for alternative hypothesis] were not significant and Bayes factors were less than 1.

### 3.4. Δ alpha band power

**Figure 5:**
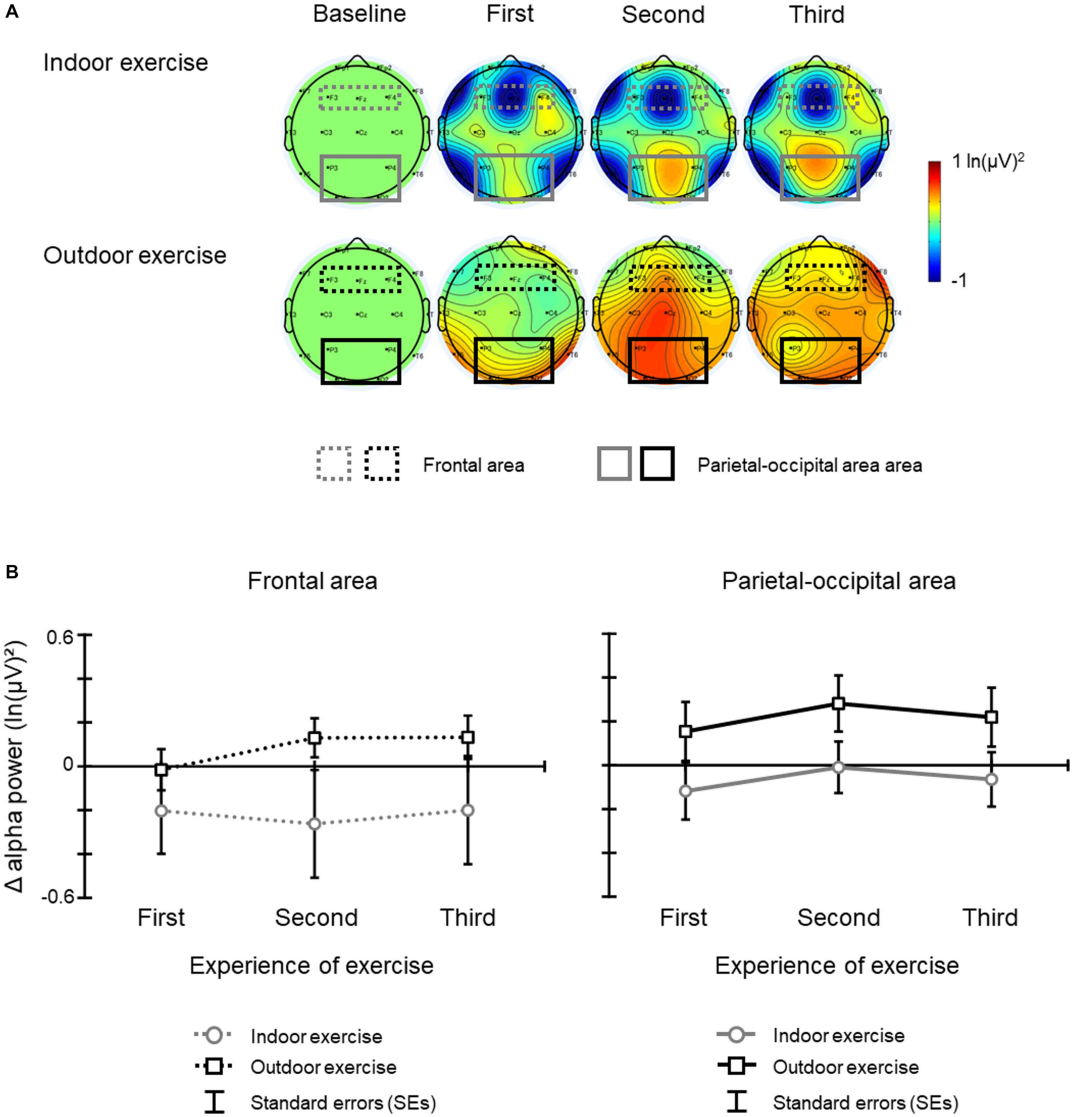
**(A)** The topographic map of the Δ alpha band power and **(B)** mean Δ alpha band power for each condition and period. The error bars indicate the standard errors (SEs) of the means across participants.

Figure 5 illustrates (a) the topographic map of Δ alpha band power, and (b) the mean Δ alpha band power at the frontal area and parietal-occipital area for each day. The results of the ANOVA and Bayesian ANOVA for mean Δ alpha band power at the frontal area revealed that the main effect of condition [*F*(1, 19) = 1.54, *p* = 0.230, η2 *p* = 0.075; BF_10_ = 0.714 indicating inconclusive evidence for alternative hypothesis], the main effect of experience of exercise [*F*(2, 38) = 0.51, *p* = 0.577, *ε* = 0.844, η2 *p* = 0.026; BF_10_ = 0.174 indicating inconclusive evidence for alternative hypothesis], and the interaction of condition and day [*F*(2, 38) = 1.50, *p* = 0.237, *ε* = 0.989, η2 *p* = 0.073; BF_10_ = 0.055 indicating inconclusive evidence for alternative hypothesis] were not significant and that Bayes factors were less than 1. In contrast, the results of the ANOVA and Bayesian ANOVA for mean Δ alpha band power at the parietal-occipital area revealed that the main effect of condition was significant [*F*(1, 19) = 4.50, *p* = 0.047, η2 *p* = 0.192; BF_10_ = 3.845 indicating moderate evidence for alternative hypothesis], and that the Δ alpha band power of the outdoor exercise condition was larger than that of the indoor exercise condition (BF_10_, *U* = 87.834; posterior odds = 87.834). The main effect of experience of exercise [*F*(2, 38) = 1.05, *p* = 0.354, *ε* = 0.871, η2 *p* = 0.052; BF_10_ = 0.248 indicating inconclusive evidence for alternative hypothesis] and the interaction of condition and experience of exercise [*F*(2, 38) = 0.01, *p* = 0.988, *ε* = 0.947, η2 *p* < 0.001; BF_10_ = 0.148 indicating inconclusive evidence for alternative hypothesis] were not significant and that Bayes factors were less than 1.

## 4. Discussion

We investigated the influence of outdoor exercise that combined a natural environment with exercise on creativity. To test this effect, we conducted an experiment to measure AUT performance, EEG for AUT, and FSS after indoor and outdoor exercise.

The HR during each exercise was over 150 b.p.m. A previous study reported that HR during exercise correlates to the RPE; RPE multiplied by approximately 10 is HR (Borg, 1982). These results show that participants performed somewhat hard to hard exercise (i.e., 14–15) under both exercise conditions.

The Δ alpha band power at the parietal-occipital region increased during the AUT after outdoor exercise compared with after indoor exercise. The Bayes factor results also showed moderate evidence, which supports this result. The alpha band power at these areas is related to the creativity of divergent thinking (Martindale and Hasenfus, 1978; Mölle et al., 1999; Fink et al., 2009; Fink and Benedek, 2014). This result shows that the cognitive activities underlying creativity are enhanced after outdoor exercise compared to indoor exercise. Moreover, this effect occurred consistently from the first day to the last day of outdoor exercise. This result suggests that the enhancement effect of outdoor exercise is not a transient effect but may occur consistently. The only difference between the exercises was whether or not they were performed in a natural environment. Our results are the first report that the combination of a natural environment and exercise consistently enhances cognitive activities related to creativity.

In contrast to the Δ alpha band power at the parietal-occipital region results, the Δ alpha band power at the frontal region did not differ between exercises. The alpha band power at these areas during the AUT is related to internal processing and top-down control (Knyazev, 2007; Fink and Benedek, 2014). Moreover, the alpha band power activity at these areas decreases when repeating the AUT, and the scores on the AUT itself (e.g., the number of ideas) decrease. This effect has been called the serial order effect (Agnoli et al., 2020). Considering this effect, we had hypothesized that the AUT scores of first day of outdoor/indoor exercise would remain at or decrease from the baseline, that these scores would not be increased through the experience of exercising outdoors/indoors three times, and that no significant difference between the AUT scores for the outdoor and indoor conditions would be observed. In fact, our results showed that AUT scores (Δ fluency, Δ flexibility, and Δ originality score) did not differ between exercises and these scores did not increase clearly from the baseline (i.e., before the exercise) after both exercises. Based on these previous studies, our results can be interpreted as having occurred because participants answered AUTs three times a day for 2 days for the baseline, which is before the exercise day, and answered AUTs repeatedly for each exercise day thereafter. Putting together results of Δ alpha band power at the parietal-occipital region, at the frontal region, and AUT scores, it will be necessary to further examine what kind of task should be used to measure creativity repeatedly in future studies.

Interestingly, Δ alpha band power after the indoor exercise was similar to or lower than the baseline (Figure 5). This result seems to contradict previous studies that reported that moderate exercise improves creativity even with indoor exercise. This difference might be due to repeated exercise and repeated AUT. Many previous studies have examined the association between short-term exercise (once or twice) and creativity (e.g., Netz et al., 2007; Oppezzo and Schwartz, 2014; Aga et al., 2021), and have not repeated the exercise and AUT as in this study. Taken together, the results suggest that the effects of fixed environments and fixed movements (indoor exercise) on creativity are short-term effects, and that the effects of outdoor exercise on creativity might be maintained even if the exercise is repeated.

Moreover, the results of the Δ AUT showed that these scores after the indoor exercise were below the baseline and decreased, whereas after the outdoor exercise scores increased slightly (Figure 3). This result seems to contradict previous studies that reported that repeating the AUT decreases these scores. This difference might be due to the context in which the AUT was repeated. The decrease in AUT scores with repetition had been shown indoors and without the exercise intervention, and the effect of outdoor exercise had not been examined (Beaty and Silvia, 2012). Taking together this result and the results of Δ alpha band power, these results suggest that factors involved in outdoor exercise might be influencing the AUT score beyond the reduction with repetition. In the results of the Δ FSS, Δ PEE and Δ AC scores increased after outdoor exercise compared with after indoor exercise. The Bayes factor results also showed anecdotal evidence. These results suggest that participants rated indoor and outdoor exercise as subjectively different. Moreover, it might be possible that the increase in Δ PEE and Δ AC scores after outdoor exercise related to the enhancement of creativity based on the EEG results. Previous studies reported that creativity was enhanced by performing free movement rather than fixed movement even with the same amount and intensity of movement (e.g., Kuo and Yeh, 2016; Murali and Händel, 2022). In our results, it might be possible that the participants’ subjectivity differed between running on a treadmill in indoor exercise and running on an outdoor course with their eyes on a natural environment. These results suggest that the effect of the combination of a natural environment and exercise influences the positive emotion and concentration of one’s own exercise, and these enhances creativity.

Finally, several points about these results need to be considered. First, the difference between outdoor exercise and indoor exercise and the factors behind it need to be clarified in more detail. In the outdoor exercise, there are changes in scenery, wind conditions, temperature, and humidity that are absent in indoor conditions. However, it is not clear which of these factors, or combinations of them, have the most influence on the results of this study. In addition, these factors might have increased the variation of movement during outdoor exercise compared to the fixed movement that occurs during indoor exercise (e.g., Kuo and Yeh, 2016; Murali and Händel, 2022). These points should be investigated in a future study. Second, the difference of HR between outdoor and indoor exercise needs to be examined. Considering the results of HR and SE, HR was lower during outdoor exercise compared to during indoor exercise. It might be possible that various aspects of the natural environment decreased the HR during outdoor exercise (e.g., Park et al., 2010); this is unclear in this study. Together with the first limitation, future research should include comparisons with outdoor exercise in less natural environments such as in an urban city with no trees around. Third, it is necessary to consider the difference of the variability of the alpha band powers between conditions in the frontal area and in the parietal-occipital area. The results of Δ alpha band power showed the variability between conditions in the frontal area but not in the parietal-occipital area (Figure 5). This difference might be dependent on the role of frontal alpha band power and the state of the subjects during the creativity task. Frontal alpha power in generating creative ideas reflects internal attention (Knyazev, 2007; Fink and Benedek, 2014). Internal attention is related to top-down control, which actively suppresses task-irrelevant information such as irrelevant sensory processing and interference information, and is also related to the degree of concentration on the creativity tasks (Sauseng et al., 2005; Klimesch et al., 2007). Therefore, the variation of Δ alpha band power in the frontal area might be related to the degree of each subject’s concentration on the task such as reflected in the AC score. Future research should examine the relationship between a subject’s state and these activities.

## 5. Conclusion

This study suggests that outdoor exercise influences positive emotion and concentration on one’s own exercise, and increases neuronal activity in brain regions related to creativity. Further research is needed to understand how this can lead to increased creativity and what factors in the outdoor environment affect creativity.

## Data availability statement

The raw data supporting the conclusions of this article will be made available by the authors, without undue reservation.

## Ethics statement

The studies involving human participants were reviewed and approved by Graduate School of Information Science and Technology’s Research Ethics Review Board under Osaka University Regulations. The patients/participants provided their written informed consent to participate in this study.

## Author contributions

TK and TM have contributed equally to this work, the study’s conception, design, and data acquisition. YT and MO designed indoor exercise and outdoor exercise. TH and YY contributed to funding acquisition and project administration. TK wrote the first draft of the manuscript and contributed to the statistical analysis. All authors contributed to reading, discussing, and revising the manuscript, and approved the final version of the manuscript for submission.

## Funding

This work was supported by MEXT “Innovation Platform for Society 5.0” Program Grant Number JPMXP0518071489. The funding sources were not involved in the study design; collection, analysis, and interpretation of data; writing of the report; or decision to submit the article for publication.

## Conflict of interest

YT and MO are employed by ASICS Corporation.

The remaining authors declare that the research was conducted in the absence of any commercial or financial relationships that could be construed as a potential conflict of interest.

## Publisher’s note

All claims expressed in this article are solely those of the authors and do not necessarily represent those of their affiliated organizations, or those of the publisher, the editors and the reviewers. Any product that may be evaluated in this article, or claim that may be made by its manufacturer, is not guaranteed or endorsed by the publisher.
